# A qualitative investigation of the allergic rhinitis network from the perspective of the patient

**DOI:** 10.1038/s41533-019-0147-5

**Published:** 2019-09-19

**Authors:** Biljana Cvetkovski, Vicky Kritikos, Rachel Tan, Kwok Yan, Elizabeth Azzi, Pamela Srour, Sinthia Bosnic-Anticevich

**Affiliations:** 1 Quality Use of Respiratory Medicine Group, Woolcock Institute of Medical Research, University of Sydney, Sydney, NSW Australia; 2 Royal Prince Alfred Hospital, Sydney, NSW Australia; 3 Sydney Local Health District, Sydney, NSW Australia

**Keywords:** Health occupations, Health services

## Abstract

Patient self-selection of over-the-counter medicines for the management of allergic rhinitis is suboptimal. The mapping of the allergic rhinitis network demonstrates that patients’ decisions with regards to their allergic rhinitis management can be influenced by up to 11 individuals/resources (alters). This study aimed to identify the role of alters within the allergic rhinitis network and identify the factors that determined their degree of influence as perceived by the patient. This research was a qualitative exploration embedded in an empirical framework and social network theory. People with allergic rhinitis were interviewed about their network and transcripts were analysed deductively and inductively. Transcripts were coded by researchers independently and then discussed until agreement was reached. Forty-one participants described the roles of 17 alters on their allergic rhinitis management. The roles of alters fell within five categories: diagnosis, medication prescription/supply/administration, medication recommendation, information about allergic rhinitis and emotional support. Participant interactions with these alters were often acute and had a long standing effect, with the participants often navigating the long-term management on their own. The significance of the influence of each alter on their allergic rhinitis management was dependent on the level of trust in their relationship, impact of the role made to the participants’ day-to-day management of allergic rhinitis and/or the participant’s beliefs. Allergic rhinitis management was fragmented and had opportunity to be improved by developing strategies, resources and policies to support self-management in collaboration with patients and health-care professionals.

## Introduction

All over the world, people with allergic rhinitis (AR) manage their symptoms with medicines that are purchased over the counter (OTC) in community pharmacies.^1–6^ In Australia, the range of medicines that are available on an open shelf, without having to consult or interact with a pharmacist, is broad and includes antihistamines and intranasal corticosteroids. While this system may provide easier access to more affordable medications, it also presents many challenges.^2^ Research investigating purchase of AR medications in the Australian setting has demonstrated that nearly 70% of people with AR self-select medication from pharmacies, with only 15% of these individuals optimally managing their AR symptoms.^2^ While these figures are concerning from a health-care perspective, people with AR express confidence in their ability to manage their symptoms,^7^ often underestimate the severity of their condition,^8^ and continue to live with an avoidably high burden of disease associated with suboptimal AR management.^6^

Medication management of a chronic disease is only a fraction of what is required for successful self-management.^9^ While there is evidence to demonstrate the benefits of self-management in other chronic diseases such as asthma and diabetes,^9–11^ the continued burden and suboptimal management of AR symptoms in the community demonstrate that AR self-management needs to be reviewed and better supported in practice.

**Fig. 1:**
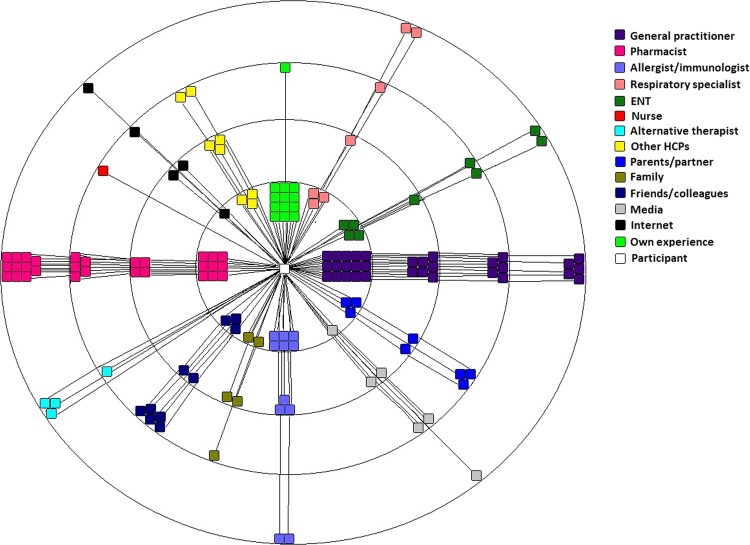
AR Network Map,^12^ Licensed under a Creative Commons Attribution 4.0 International License

However, prior to developing and optimising AR management strategies, we need to understand the influences behind patients’ AR decision making and behaviours to identify where the opportunity to improve AR management lies. Recent research exploring patient perspectives of AR management provide some insights. Specifically, recent data show that, while on the surface people with AR seem to be making their own decisions with regards to AR medication selection in the pharmacy,^3^ research utilising the principles of social network theory has identified that their decision-making is embedded within an identifiable network.^12^ The AR network was developed utilising the principles of egocentric social network theory, which identifies connections and relationships with people and resources that influence health behaviours.^13^ The previously published AR network appears in Fig. 1.^12^ The AR network map is a visual representation of the people and resources (alters) that were identified by people with AR as having an influence on their AR management. Alters were plotted on the map to show that those perceived to have greatest influence are closest to the centre of the map and those with least influence are furthest from the centre.

While this research uncovered a densely populated network identified by people with AR as being of influence on their AR management, care must be taken in its interpretation as the map alone does not reveal the particular role of each alter in the patients’ AR management and there are several inconsistencies between what the map suggests and what the empirical evidence of patient behaviour in the community pharmacy suggests. For example, while the AR network map shows that general practitioners (GPs) and pharmacists to be most influential in the patients’ AR decision making, only 14% of patients purchasing AR medication in the pharmacy stated that their decision was based on the advice of a health-care professional (HCP).^3^ Clearly, there is a gap in our understanding of what role AR networks play in management and how they drive patients’ decision making. Therefore, this study aims to use qualitative enquiry to explore the patients’ perceptions of the roles of alters within the AR network and to understand their reasons behind their perceived level of influence on their AR management.

## Results

Forty-seven people with AR who completed their AR network map were invited to take part in this study and provided informed written consent. Forty-one completed the interview, with the remaining seven not completing the interview citing time limitations. Following data collection, all forty-one interviews were coded. Following the 21st interview, no further original codes were identified. Participants were aged between 18 and 86 years (median 38 years, interquartile range 30–54) and 67% were female. Eighty-three percent were from metropolitan Sydney and 17% from regional New South Wales.

### Roles of alters within the AR network

The roles of alters within the AR network as described by participants can be grouped within five categories; diagnosis, medication prescription/supply/administration, medication advice, information about allergic rhinitis and emotional support. The fact that some alters were described to have multiple roles means that some appear in more than one category and that more than one type of alter can perform the same role with respect to AR management.

#### Diagnosis

GPs, allergists and immunologists, ear nose and throat surgeons, respiratory specialist, an optometrist, a dentist, family and friends and the participants’ own experience were mentioned to have a role in the ‘diagnosis’ of AR. In some instances, the HCP observation of the physical manifestations of AR served to confirm a previous ‘diagnosis’ or suspicion of allergic sensitivity. The process of diagnosis was not always straightforward or well articulated, and in most instances, the participants reported it to be based on history taking of symptoms and their patterns and observations of the physical manifestations associated with AR, most often during a consultation outside of the context of AR. The use of skin prick tests for diagnosis were only mentioned with respect to consultations with allergists and immunologists (terms that were used interchangeably by the participants). In addition to providing a diagnosis themselves, GPs were identified for their role in referral to a specialist when required. Participants who nominated respiratory physicians within their AR network as a diagnostician also had comorbid asthma and said the consultations with them were predominantly for their asthma.

There were also a subset of participants who had never seen a HCP about their AR and had self-diagnosed their AR based on symptoms such as sneezing and watery eyes, particularly if in response to an identifiable trigger such as animal dander or during a particular time of year often associated with AR. Self-diagnosis was also more prevalent if a family member also had AR and participants had identified similarity in their symptoms and experiences. Similarly, family and friends with AR were likely to offer a ‘diagnosis’ where they had made observations of symptoms and patterns they were suffering themselves.

“In the first instance, I’ve got a GP who referred me to an allergist.” (P57)

“When I was a child, my GP noticed I was always sniffling, coughing and its wasn’t just fixed with Ventolin.” (P42)

“My oldest brother has the same sort of thing as me.” (P44)

#### Medication prescription/medication supply/medication administration

The role of the GP in prescribing medication was commonly reported. This included prescriptions for intranasal corticosteroids and antihistamines. If participants were able to access the prescribed medications OTC, they would not return to their GP for repeat prescriptions. In most circumstances, participants reported that AR was not the primary reason they had visited their GP for a consultation. This was often the case where the participant had comorbidities and was visiting their GP to have those addressed and would mention their AR at the end of a consultation. This was especially the case where there was an existing, long-term relationship between the GP and their patient. Participants who did visit a GP for a prescription often visited a medical centre and consulted GPs who they had no prior relationship with. If repeat visits were required to obtain repeat prescriptions, they would not necessarily visit the same GP. Participants also reported isolated interactions with their GPs with regards to their AR. AR was not something that they revisited their GP for and there were no reports of review consultations. The participants did report that they held on to the advice they received at consultations for many years and did not question the need for a review or change in management strategy.

“I think when I found out I didn’t need to see the GP anymore, I’ve just been to the pharmacist.” (P8)

“It’s been a while since my GP recommended that. I’ve never gone back for a follow up.” (P31)

“I had it on prescription when I was a child in [Europe] and since I came to Australia I just buy it.” (P55)

Allergists and immunologists were identified for their role in prescribing immunotherapy. Some of the participants described being prescribed multiple courses of immunotherapy. Apart from requiring repeated immunotherapy, few participants reported any long-term follow-up with their allergists/immunologists.

“I was advised at the time to just do short courses of those and I didn’t end up going back for -he didn’t need to see me again. So I didn’t actually have it followed up. (P42)

Respiratory physicians were identified as having a role in prescribing treatment for AR. This was particularly during circumstances where the participant had an established relationship with respect to their comorbid asthma.

“I like him[respiratory physician] because he doesn’t believe in operations, whereas most other guys want to do other big things to your nose.”……..“I don’t really trust anybody else. I trust [respiratory physician] with my life. He has helped me enormously over the years and I don’t even really trust the GP.” (P33)

Some participants reported that they view the role of the pharmacist as a medication supplier. They reported knowing exactly what medicines they want and that they do not need advice or recommendation from a pharmacist. Upon self-selecting their medicines and making the purchase, they report being asked about whether they have used the medicine before to which they reply ‘yes’ and have no interest in discussing it further.

“I just go in there and buy the stuff half the time and don’t even talk to them. I go in and I’ll be getting a Seretide script made up and I’ll get some Zyrtec or something.” (P2)

A practice nurses was nominated within the AR network for their role in the administration of immunotherapy injections.

‘A practice nurse helped with the injections.’ (P6)

#### Medication advice

Pharmacists, family and friends and participants’ ‘own experience’ were most commonly reported for their role in providing medication advice for the management of AR symptoms. (The GP’s role in providing medication advice, which is more formal and structured, is described earlier in their role as prescribers).

Pharmacists were often consulted with regards to recommending medications for AR. Consultations with the pharmacist or their staff were done at regular intervals during their repeat visits to the pharmacy for AR medicines, most specifically about whether any new treatment had become available. Participants also reported that pharmacists’ advice on medication encompassed safety concerns with pharmacists checking for potential drug contraindications with AR medications purchased over the counter.

Participants’ own experience involved experimenting with OTC nasal products. On the rare occasion, a participant would identify a non-medication-related strategy that they had discovered to ease their AR symptoms, such as swimming. Participants would also rely on recommendations from their family and friends, especially if they were also AR sufferers and had recommended a particular product. Family and friends were identified for recommending medicines to treat AR symptoms and organising medical investigations/consultations. Recommendations made by a family member were especially held in high regard as there was a belief that familial people would have the same response to treatments.

Participants reported exploring AR management with a variety of alternative therapists. In some instances, participants felt their AR symptoms improved while others did not. Participants reported consulting an alternative therapist when they felt traditional therapies for AR were not successful. Other HCPs identified on the AR network map included a neurologist, dermatologist and optometrists for making treatment recommendations for AR symptoms during consultations for their area of specialty.

“I just self-select and I don’t really consult anyone else. That’s just been working for me so far. It hasn’t really been 100% working, because it’ s still happening.” (P56)

“It’s trial and error. I just know-you know what works for yourself.” (P36)

“He[the pharmacist] was really good. He was quite concerned about things; if any drugs that contraindicated. He used to check them.” (P9)

“[Hay fever] affects my eyes so badly and I wear contact lenses my optometrist is probably a big one[influence].” (P4)

“[My husband] is an expert and he’s been able to just find a tablet for me that is non-drowsy and it does seem to work quite rapidly, even though it does take an hour and a half.” (P30)

“I’ve got more faith in them [friends rather than health care professionals].” (P10)

#### Information about AR

Information regarding AR management was both actively sought and passively received by participants. Participants reported receiving and searching for information from pharmacists, family and friends, internet and media. Pharmacists were described as having a role in providing information about AR management beyond recommendations for medication. Participants valued the pharmacist providing instruction on correct use of an intranasal inhaler device as well as discussing minimisation of exposure to allergens. Pharmacists were perceived to have time available to be able to provide information to their patients. Family and friends were also noted to be a source of information about AR management, most particularly about medications. Many participants reported being told by family and friends that they can develop a tolerance to one type of antihistamine and that they should alternate therapy with more than one type.

The alter titled ‘Media’ encapsulates television news and current affairs programs, newspapers, pamphlets and advertisements for commercial products. Participants received information about AR from these sources both passively and actively. Participants acknowledged the difference between commercial advertisements and information provided by experts in the field but were always interested in hearing about ‘new’ information, irrespective of the source. Not all information received would always be acted upon immediately but sometimes reflected upon at a later time point.

The internet was placed in the AR network as a source of information that participants used proactively to educate themselves about AR; however, they had varied perspectives of the reliability of information available. Participants had actively searched for information about AR treatments for instructions on how to use their medicines. Otherwise, very few participants reported actively searching for AR information on the internet.

“The pharmacist [checked nasal spray technique] did and I think I’m using it right. The nasal spray-the chemist-actually the local chemist where I work was the first guy who said you’re supposed to point the tube between your eyes when you put it up your, at an angle.” (P25)

“I ended up doing a search on hay fever on medications or something like that……….Well the reason I was looking at it because I was just looking and trying to pick up on-I was checking about the correct way to take medication.” (P21)

“I’ll always keep a casual eye out in the press, in the media, the news like for pollen index or any new medical advances.” (P1)

“Advertising [in response to why a particular medication was selected].” (P20)

“Papers written by experts or persons in the field, the doctorate field, that I take any notice of. Anyone that has written stuff that is not associated or trained in that field, no. It’s with a grain of salt.” (P9)

“I have seen some pamphlets [in the pharmacy] and I’ve taken them away and read them. It’s kind of like they all say the same thing whatever.” (P33)

#### Emotional support

Overall, very few participants described needing emotional support from their AR network. In the few instances that this was mentioned, emotional support was provided by family and friends, (especially if they too had suffered from AR). Emotional support involved the alters providing empathy when the participants were not feeling their best due to their AR or feeling frustrated during a lengthy course of immunotherapy.

“[Mum] had the desensitisation. There was another girl in the office here … with me that - she went through the desensitisation process. I discussed it with her. An uncle that gets hay fever really bad as well. My wife, anybody that’ll listen on a snotty, [expletive] day.“ (P13)

### Degree of influence within the AR network

The factors that determined the participants’ perceived degree of influence within the AR network were unique to each participant and their experiences with AR. These determining factors were not necessarily mutually exclusive and more than one may have been a determining factor of influence within the individual participant’s AR network.

#### Perceived impact on day to day management

The perceived impact of the role of the alter on the day-to-day AR management determined the degree of influence within the AR network. This was independent of when or how frequently the contribution was made. If the participants’ current AR management was impacted, they were regarded to have more influence within the AR network.

“I would place the immunologist that recommended the injections at number one, because that helped.” (P31)

#### Trust and confidence

Trust of HCPs was determined by the participants’ preconceived ideas of the role of that HCP and their competence, i.e. HCPs who met the participants’ expectation were trusted. Participants with existing long-term relationships with HCPs reported the highest level of influence within their AR network. HCPs who were seen as being proactive earned the trust of participants. In some instances, parents/partners and pamphlets were reported to be more influential than HCPs with whom the participant had had an unsatisfactory experience.

“Yeah, I just find that they just have better overall knowledge about medications and stuff.” (P15) (pharmacist strongest influence)

“It was actually my GP who brought it up because she suffers from asthma and hay fever and she just said she’s noticed when she gets a hay fever attack her asthma is aggravated, I guess. Yeah so she actually asked me to try the nasal spray to see if it was effective and if it helped me because its helps her and to see if there was any change in my asthma.” (P23) (GP second highest level of influence)

#### Participant beliefs

If the participant believed in medicines and the role of the HCP, then HCPs had a stronger influence within the AR network than non-HCPs, such as their family and friends.

Non-HCPs such as family and friends had a stronger influence within the AR network if the participant did not believe in HCPs and the effectiveness of medicines. These participants expressed that they would implement a friend’s recommendation if they have had a positive experience from it because they feel they had similar values with regards to what they are looking for in terms of AR relief.

“I do have a regular pharmacist but they are not particularly helpful. They’re not helpful but… they did offer me the Nasonex and I think my mum had some and she gave it to me at the time and then I started using it and it just wasn’t working fast enough.” (P25) (pharmacist lowest degree of influence)

## Discussion

The aim of this research was to qualitatively explore the patient’s perceptions of the roles of alters within their AR network and to understand what determines their perceived degree of influence. While we already knew that people with AR obtain advice from a wide range of sources, we now know that the advice being provided is suboptimal, fragmented and not supported with follow-up review. This research demonstrates that, although participants identified themselves as having AR, very few described an official diagnosis and were making medication management decisions based on information provided many years ago without follow-up review. Overall, it is apparent that people with AR continued to be burdened by their symptoms, as they venture widely for guidance on AR medication. Unfortunately, in this era of patient self-selection for AR medications, patients are clutching at fragments of information obtained from a range of sources and making decisions without taking opportunity for assessment and evaluation. Our research demonstrated that, in the current environment of medication self-selection, there is an urgent need for an AR self-management strategy to be implemented in primary care.

This qualitative exploration of the AR network map identifies several issues with the current management of AR in the community that were otherwise not apparent on the AR network map independently. Diagnosis of AR is suboptimal in practice with many people self-diagnosing their condition. While GPs were reported for their role in diagnosis, reports of comprehensive diagnosis of AR were not available. This data confirms that AR diagnosis is a continued challenge in primary care^14–17^ and that inaccurate diagnosis may be a factor in patient disillusionment with the management of their symptoms. Looking beyond the issues of diagnosis, HCPs were readily dispensing medication advice, yet very few opportunities for follow-up were reported and participants often continued to implement this advice long term without review. Participant reports of using their ‘own experience’ to determine their AR management further confirms the limited amount of review consultations with HCPs. Stand out reports of follow-up included interactions with the pharmacist during repeat purchases of medications and requests for anything new, but these were missed opportunities in that extensive review and assessment of current management was not described.

In addition to the identification of these systemic issues in the current management of AR in primary care, several patient factors were identified. Our research demonstrated that, there is no ‘role’ that is most significant in influencing a patient’s AR management. The degree of influence within the AR network is determined by three factors: the impact of the role of the alter on the patient’s day-to-day management of AR, the patient’s trust and confidence in the alter, and the patient’s health beliefs. These results are in line with established knowledge that patients’ beliefs influence behaviour^9^ and with well-known behaviourist approaches, including Cognitive Theory and the Health Belief Model.^18,19^ However, the significance of this outcome for practice is to highlight that more comprehensive research into the impact of the health beliefs of people with AR on their clinical outcomes is required. Optimising the management of AR in the community has always been challenging due to perceptions of triviality among the community and patients’ ‘lay expertise’^20^; however, most chronic diseases have patient management behaviour that is determined by their beliefs.^9^ The difference lies in the strategies developed to recognise and accommodate these beliefs within their management and interactions with HCPs. While AR management goal-setting strategies incorporating patient beliefs have been demonstrated to be successful,^21^ there is little evidence of their use in the community. HCPs supporting AR self-management need to be provided with tools to evaluate patient beliefs about AR and its management and adapt AR management strategies to accommodate these beliefs and we may need to look towards studies in asthma that explore patient beliefs and attitudinal clusters.^22,23^

A qualitative exploration of the AR network from the perspective of the patient has not been conducted before. This research methodology has only relatively recently been used to explore patient-reported networks in asthma^24,25^ and was chosen to explore AR management because the burden of AR on the community continues to be high, despite the availability of gold standard guidelines, pharmacological therapy and extensive research into HCP and patient perspectives.^26–29^ Similarly to the studies exploring asthma networks, this research identified patients accessing a broad range of resources to make decisions about their AR management, but unlike the asthma networks, people with AR reported more reliance on their own experience and isolated interactions with HCPs. Self-selection of medication is only one part of the dynamic self-management puzzle.^9^ Self-management requires supporting people to understand their condition, being able to monitor it and take appropriate action.^9^ Self-management requires continued collaboration among HCPs and the patient and must take into account the patients’ beliefs in order to be successful.^9^ Additionally, the HCPs providing the support for self-management must be supported with policy and protocols.^10,30,31^ This research has identified that several areas of AR management need to be targeted in order to optimise AR self-management and both HCPs and people with AR are in need of support.

Although this study has some limitations, they themselves have demonstrated the need for further research to target AR self-management in primary care. A significant limitation of this study is the self-reported diagnosis of AR among the participants, many who described their AR as self-diagnosed. The impact of this limitation is that the description of the roles of alters within the AR network are reflective of the population of people who manage their symptoms within the community setting believing they have AR and not those who have been formally diagnosed with AR. While future research into the AR network would seek to accurately recruit those diagnosed with AR, the population of this study has demonstrated that there are many people who believe they have AR and that provisions need to be made to ensure accessibility to diagnosis to optimise management of symptoms. A broader recruitment of future participants would address this study’s limitation associated with its sample size and recruitment pool. A larger and broader sample would add to the research to determine whether there is any variation with regards to geography, access to health care and socio-economic status and health beliefs.

Further research into the AR network should explore possible variations among different AR phenotypes, existence of comorbidities, patient personalities and differing health beliefs.

In conclusion, this qualitative exploration of the AR network has demonstrated that people managing their AR are not as well supported as the AR network map may suggest. AR management in the community has opportunity to be improved with the development of strategies, resources and policies to support AR self-management in collaboration with patients and HCPs.

## Methods

### Study design

This research is the second part of a two-part study exploring the AR health network from the perspective of the patient. Part One was the quantitative depiction of the AR health network^12^ (Fig. 1). This research utilises qualitative research design and interviews with people with AR about their AR network. The basis of this research was embedded within an empirical framework and egocentric social network theory,^12,24^ where we used the quantitative information of the AR network to further enquire about the roles of the alters in relation to the patient, being the centre of the network. This study was approved by the University of Sydney Human Ethics committee. Methods were performed in accordance with consolidated criteria for reporting qualitative research regulations and guidelines.^32^

### Participant eligibility

To be eligible to participate, participants were aged ≥18 years, able to speak English, identified themselves as having AR and had participated in the Part One of the study, detailing their AR health network map.^12^

### Participant recruitment

Participants were recruited for the larger study by advertisements placed on the website and Facebook page of the Woolcock Institute of Medical Research, letters of invitation sent to the Woolcock’s volunteer database, as well as through expressions of interest following media reports of our study appearing on the Sydney metropolitan evening news bulletin. People who contacted the research team expressing interest in participating were screened for eligibility and required to sign informed consent prior to commencement in the study.

### Sample size

The sampling frame was all people with self-reported AR who had participated in Part One of the study. All participants of Part One were invited to participate in the interview.

### Data collection

Data collection took the form of an interview utilising an empirical framework based on the AR network^12^ and name interpreter component of egocentric social network theory.^33^ The interview guide appears in Table 1. Participants had the option of being interviewed in person or over the telephone. All interviews were conducted by B.C. Audio was recorded on a digital recording device and interviews were transcribed verbatim. Prior to commencement of the interview, participants were informed that their responses would be recorded and that they would be de-identified upon transcription. Written consent was obtained prior to commencement of the study. Data were stored in accordance with the University of Sydney Human Ethics policies.

**Table 1:** Semi-structured interview guide

Name interpreter questions	Tell me about how you discuss allergic rhinitis with your contacts?	What impact does your contact have on your allergic rhinitis management?	How has your contact influenced your allergic rhinitis management?
Level of influence questions	How important is your contact to your allergic rhinitis management?	Why do you feel your contact has such an impact on your allergic rhinitis management?	What determines how important your contact is to your allergic rhinitis management?

### Data analysis

This study utilised a combination of deductive and inductive qualitative analytical approaches. The deductive analytical approach involved searching for terms within the interview transcripts that represented pre-determined themes and concepts based on the previously documented AR network^12^ and the literature on AR management.^34^ These themes included the list of alters within the AR network and the traditional roles that have been previously documented (e.g. ‘general practitioners’ and ‘diagnosis’) within the AR literature. The inductive analysis of the transcripts consisted of the identification of concepts within the transcripts that were not searched for but were found to emerge from the data. The researchers (authors B.C., R.T., E.A., P.S. and S.B.-A.) read through the transcripts and identified statements that represented participant’s descriptions of ‘roles’, ‘importance’, ‘placement’ or ‘positioning’ of influences within the AR network as well as statements regarded as significant with regards to the role of alters that had not been pre-determined (coding). Following independent coding (by B.C., R.T., E.A., P.S. and S.B.-A.) of the transcripts, two meetings were held, with B.C., R.T., E.A., P.S., S.B.-A. and V.K., to discuss the findings. Consensus on the codes was established within these two meetings. B.C. noted the final discussion among the researchers and consolidated the agreed codes and supporting quotes from the transcripts. Three further meetings were held between B.C. and S.B.-A. to discuss representation of these results for this manuscript. All authors read and approved the final manuscript.

ATLAS.ti and nVivo (based on each researchers’ preference and familiarity) were used to facilitate the qualitative analysis by providing an organisational structure with which to document the themes and the evidentiary codes.

Participants were not given the opportunity to provide feedback on the findings but were able to request a summary of the results.

### Reporting summary

Further information on research design is available in the Nature Research Reporting Summary linked to this article.

## Supplementary information

Reporting Summary

## Data Availability

The authors declare that data supporting the findings are available within the manuscript and additional participant quotes are available upon request. Permission to share entire interview transcripts was not obtained from the participants.

## Supplementary information

**Supplementary information** accompanies the paper on the *npj Primary Care Respiratory Medicine* website (10.1038/s41533-019-0147-5).
